# Swimming With A Hundred Year Old Snapping Turtle

**DOI:** 10.3201/eid1312.079999

**Published:** 2007-12

**Authors:** Freya Manfred

I spy his head above the waves,
big as a man’s fist,
black eyes peering at me,
until he dives into darker, deeper water.
Yesterday I saw him a foot from my outstretched hand,
already tilting his great domed shell away.
Ribbons of green moss rippled behind him,
growing along the ridge of his back
and down his long reptilian tail.
He swims in everything he knows,
and what he knows is never forgotten.
Wisely, he fears me as if I were the Plague,
which I am, sick unto death,
swimming to heal myself in his primeval sea.

